# Supplementary material for: Identification of Diverse Stress-Responsive Xylem Sap Peptides in Soybean
Source: Int J Mol Sci. 2022 Aug 3;23(15):8641. doi: 10.3390/ijms23158641 (PMC9369194; doi:10.3390/ijms23158641)
Supplement: Supplementary file 1 [file ijms-23-08641-s001.zip › Figure S1-S7.pdf]

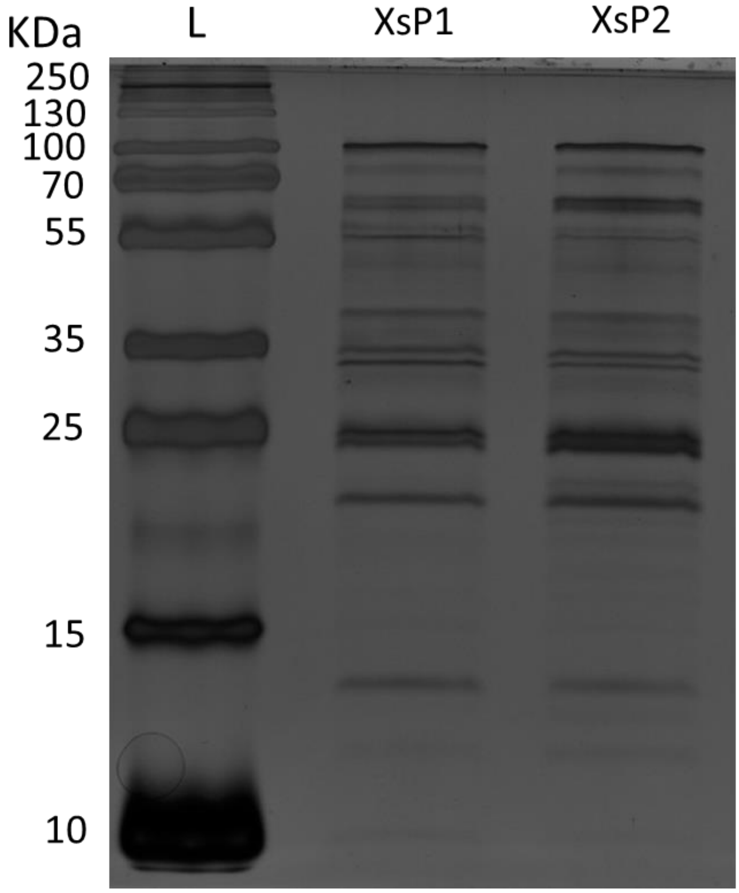

Figure S1. SDS-PAGE of soybean xylem sap proteins. Gel was stained by silver staining method. L: PageRuler™ Plus Prestained Protein Ladder (Thermo Scientific). XsP1,2: soybean xylem sap proteins from two independently collected xylem sap samples.

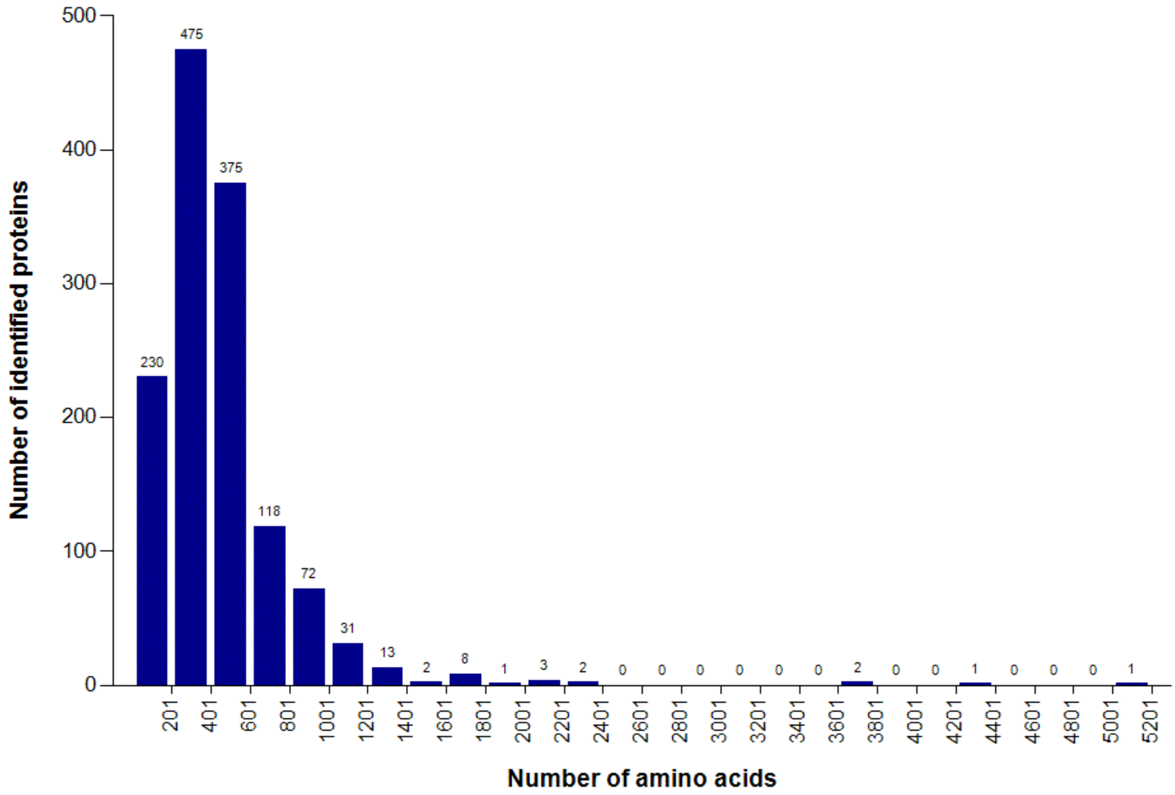

Figure S2. Protein length distribution of identified proteins from soybean xylem sap peptidome LC-MS analysis. The number of protein count are indicated on each column. A total of 1334 proteins were identified from the MS data.

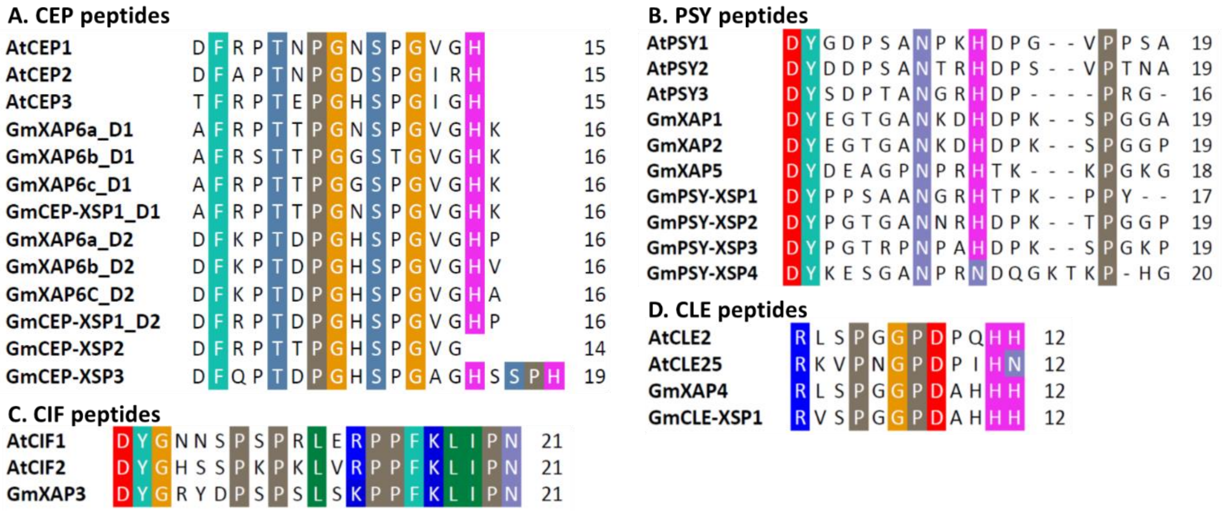

Figure S3. Multiple sequence alignment of the proposed final peptide domains of soybean xylem sap peptides with representative *Arabidopsis* peptide family members. The peptide sequences are categorized based on peptide family classification: (A) CEP peptides, (B) PSY peptides, (C) CIF peptides and (D) CLE peptides. Colored characters indicate the identical or similar amino acid residues. The total number of amino acids of sequences are indicated on the right.

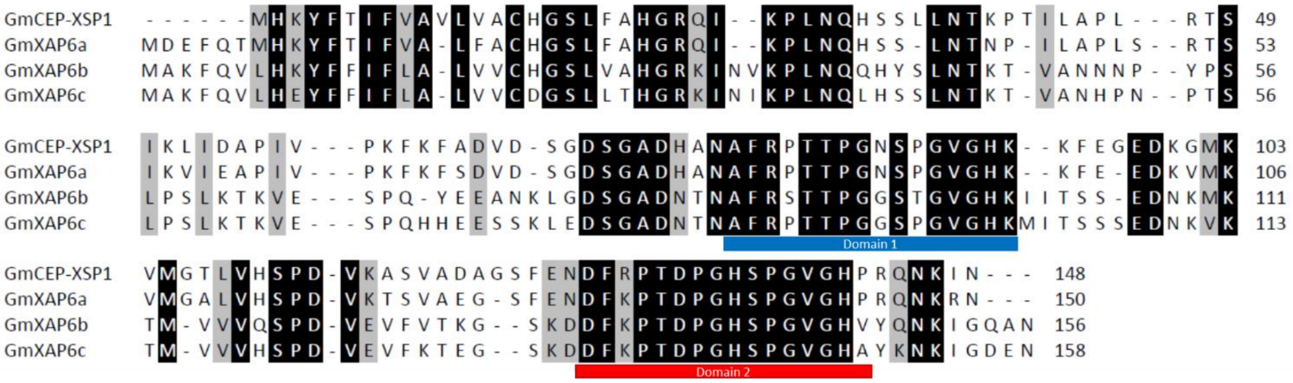

Figure S4. Multiple amino acid sequences alignment of precursor polypeptide of GmCEP-XSP1 and GmXAP6a-c. Amino acid sequences encoding the domain 1 peptides are underlined in blue and amino acid sequences encoding the domain 2 peptides are underlined in red. Identical amino acids are highlighted in black and similar amino acids are highlighted in grey.

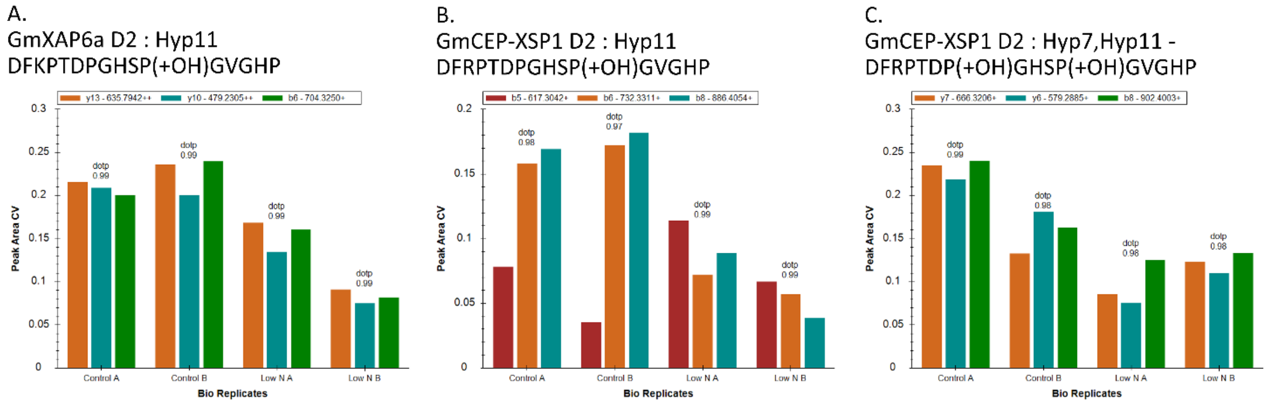

Figure S5. The mean coefficient of variance (CV) values of transition peak areas in parallel reaction monitoring (PRM) assay of peptides (A) GmXAP6a:Hyp11, (B) GmCEP-XSP1:Hyp11 and (C) GmCEP-XSP1:Hyp7,Hyp11 between soybean xylem sap under normal(control) and nitrogen deficiency (Low N) conditions. The CV values of each transition was calculated within the technical replicates (n=3).

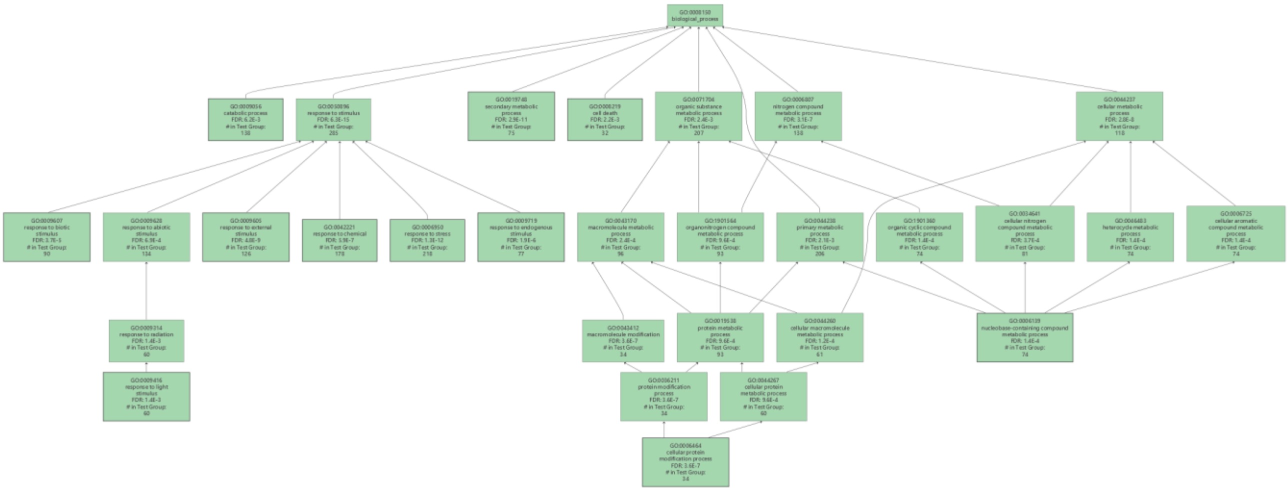

Figure S6. Hierarchy tree of enriched GO terms of 428 differentially expressed proteins of CEP1ox hairy root proteome. The enriched GO terms were filtered with conditions of having FDR <0.01 and at least 20 matched proteins. The number of matched proteins for each GO terms are indicated in "# in Test Group".

A

CLE-XSP1 hyp4	RVSPGGPDAHHH
CLE-XSP1 hyp7	RVSPGGPDAHHH
CLE-XSP1 hyp4,7	RVSPGGPDAHHH

B

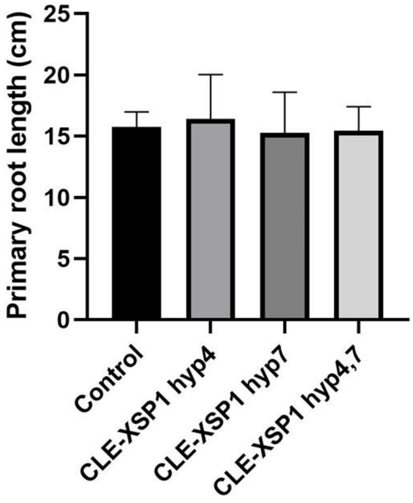

Figure S7. Biological activity of modified CLE-XSP1 synthetic peptides. (A) Modified peptide sequences of CLE-XSP1 (hyp = proline hydroxylation, Underlined = hydroxylated sites). (B) Primary root lengths of soybean seedlings after 7 days treatment of CLE-XSP1 peptides. To assess the peptides' biological activity, 4 days old soybean seedlings (n=20) were cultured with or without 1µM peptides in nutrient solution for 7 days, primary root length of treated plants was then measured. Statistical analysis by student's t-test showed that no significant difference could be observed in the treated plants. Error bars indicate the standard deviation.
